# Tumor thrombus formation in the right common iliac vein after radical proctectomy in a patient with rectal cancer: a case report

**DOI:** 10.1186/s12893-022-01768-9

**Published:** 2022-08-29

**Authors:** Jun Ma, Yaming Zhang, Chaoping Zhou, Shuqiang Duan, Yan Gao

**Affiliations:** 1 Department of Surgical Oncology, Anqing Municipal Hospital, No. 352, Ren-Ming Road, Anqing, 246000 Anhui People’s Republic of China; 2 Department of Pathology, Anqing Municipal Hospital, Anqing, 246000 People’s Republic of China; 3 Department of Anqing Oncology, Municipal Hospital, Anqing, 246000 People’s Republic of China

**Keywords:** Rectal cancer, Metastasis, Tumor thrombus, Chemoradiotherapy

## Abstract

**Background:**

Intravascular tumor thrombi are mainly found in patients with liver cancer or renal carcinoma but rarely occur in those with rectal cancer.

**Case presentation:**

This is a case report of a 58-year-old woman with a swollen right lower extremity 14 months after radical resection for rectal cancer. Although ultrasonography indicated the presence of deep venous thrombosis (DVT) located in the right common iliac vein, interventional angiography showed that a circular mass, considered a tumor thrombus, was located in the right common iliac vein. The tumor thrombus was cured by interventional therapy, and the pathological report confirmed that the metastatic tumor thrombus originated from the rectal cancer. The patient underwent concurrent chemoradiotherapy and systemic therapy. However, right lung, retroperitoneum, and 2nd sacral vertebral metastases were discovered during follow-up.

**Conclusion:**

The correct diagnosis of a tumor thrombus and its differentiation from DVT can prevent incorrect treatment and prolong the survival of patients with rectal cancer.

## Background

Venous tumor thrombi are mainly found in patients with liver cancer, renal carcinoma, adrenal tumors and Wilms tumor. Tumor thrombi mainly occur in the inferior vena cava(IVC) and renal vein [1–4].However,a venous tumor thrombus is a rare finding in colorectal cancer, seen in only 1–2% of these patients [5].In patients with rectal cancer, tumor thrombi are mainly located in the inferior mesenteric vein(IMV);they are less likely to be found in the IVC and internal iliac vein, and are almost never found in the common iliac vein[5–7].This study reports a rare venous tumor thrombus located in the common iliac vein after radical resection for rectal cancer, and discusses the diagnosis and therapy associated with this case.

## Case presentation

A 58-year-old Chinese woman who underwent radical proctectomy because of rectal cancer 14 months prior experienced sudden swelling in the right lower limb for 4 days. Deep venous thrombosis (DVT) of the lower limb was diagnosed by primary ultrasonography, but a tumor thrombus of the right common iliac vein was finally confirmed by interventional angiography and biopsy.

**Fig. 1:**
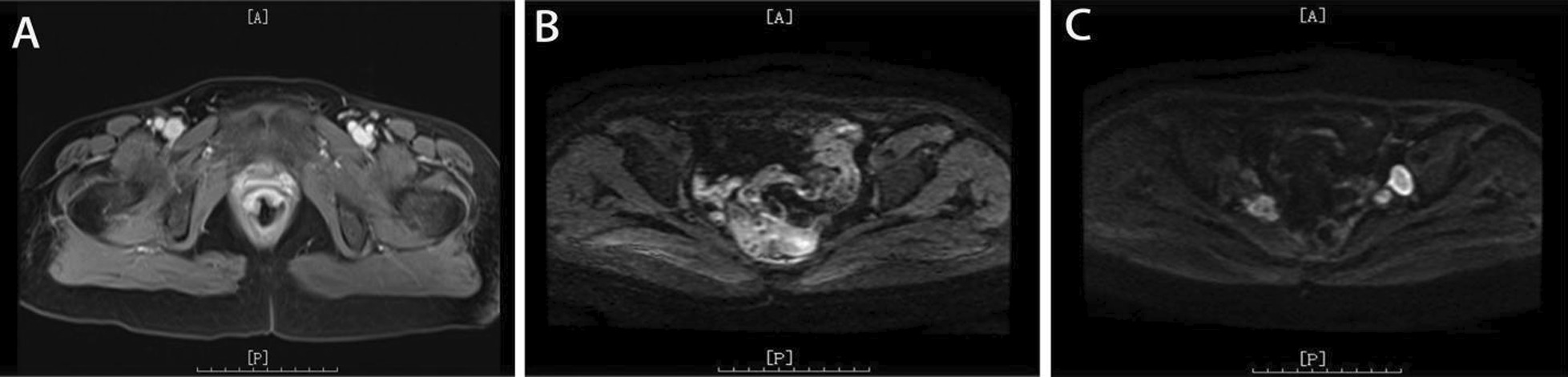
**A** MRI showed that rectal lesions were enhanced before radical rectal resection. **B** No recurrence or metastasis was found on MRI during follow-up. **C** MRI showed that the lateral lymph nodes were enlarged before chemoradiotherapy

In April 2020, the patient received a diagnosis of rectal adenocarcinoma. The patient had no history of hypertension, diabetes or heart disease and had no family history of cancer. Magnetic resonance imaging (MRI) showed that the lesion was located in the lower part of the rectum, and there was no lateral lymph node metastasis in the pelvis (Fig. 1A). The preoperative clinical stage was cT3N + M0. The patient refused neoadjuvant chemoradiotherapy due to economic reasons.

Laparoscopic-assisted abdominal-perineal resection (APR) was performed on April 22, 2020. Postoperative pathologic examination showed stage IIIA, moderately differentiated, KRAS mutated (exon 4) adenocarcinoma with no lymph node involvement (0/12) and a tumor deposit in the rectal mesentery. The circumferential resection margin (CRM) was negative (2 mm). Immunohistochemical marker results were as follows: CK (+), HER-2 (−), p53 (+), Ki-67 (approximately 65%), MSH2 (+), MSH6 (+), MLH1 (+), PMS2 (+), Syn (−), and CgA (−).

Between May 2020 and October 2020, the patient continued adjuvant chemotherapy after surgery, completing eight cycles of oxaliplatin and capecitabine (CAPEOX regimen). The patient refused postoperative radiotherapy due to a fear of radiation-related complications. No metastasis was found in the latest postoperative follow-up on November 16, 2020 (Fig. 1B).

On June 21, 2021, the patient presented swelling of the right lower limb with dark skin for 4 days. The right lower limb was highly swollen, the skin tension was tight, and the tenderness was positive. The temperature and color of the skin were higher and darker than those of the opposite side, respectively.

**Fig. 2:**
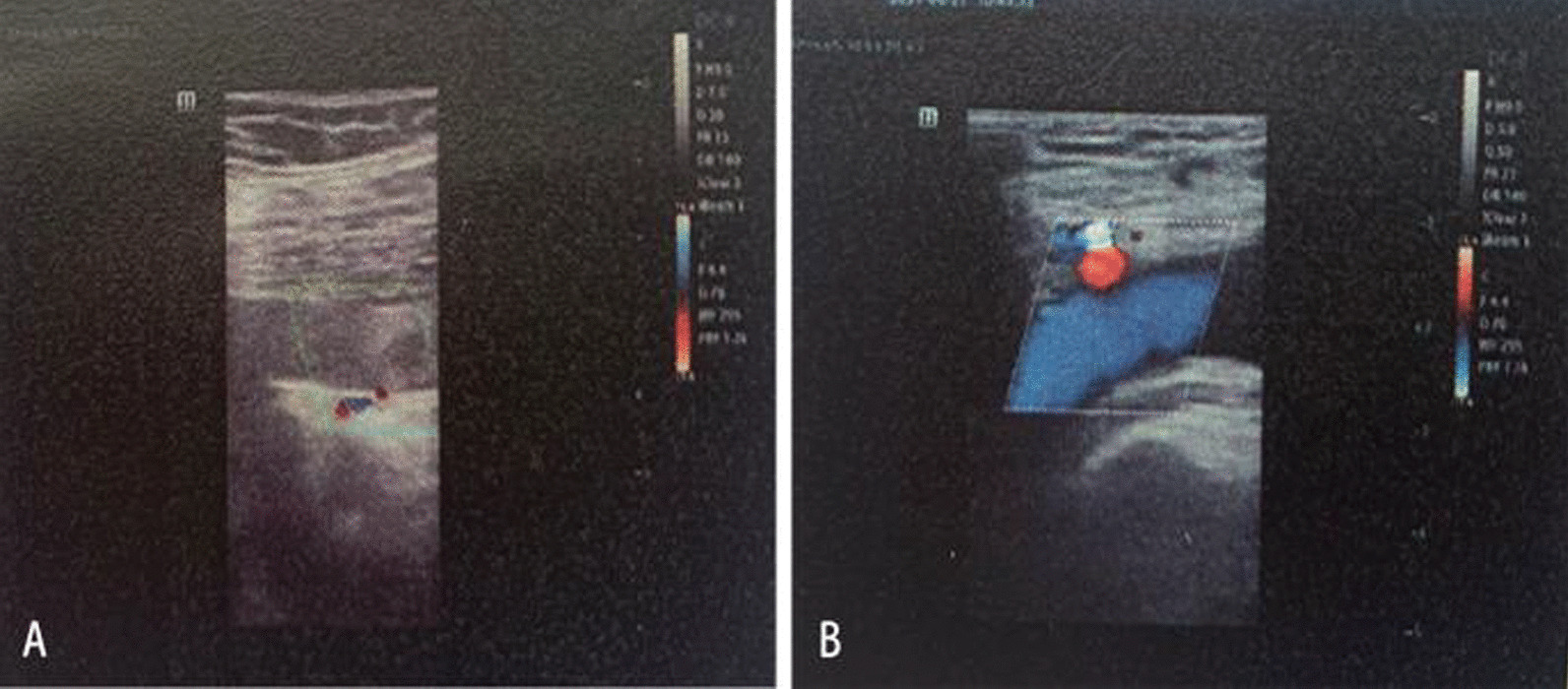
**A**, **B** Primary ultrasonography indicated the possibility of deep venous thrombosis (DVT) of the right lower extremity

**Fig. 3:**
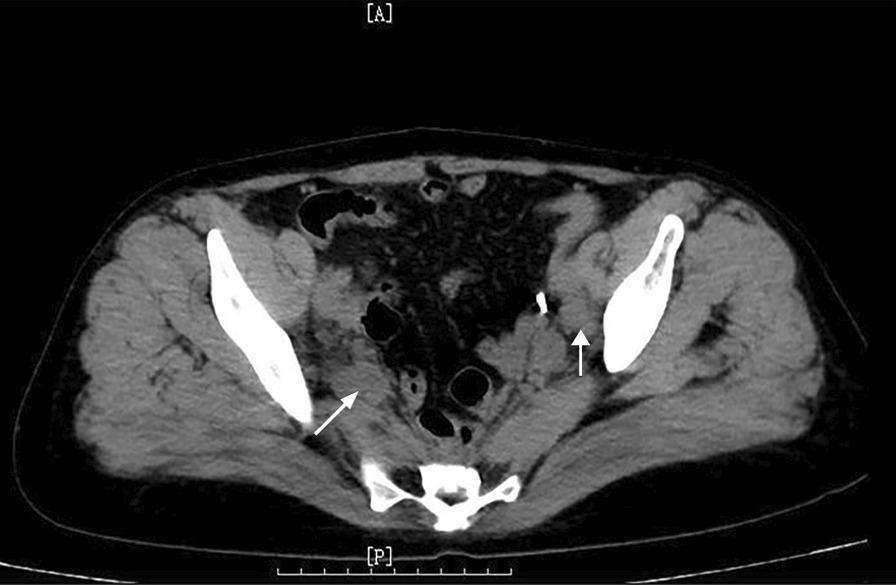
Pelvic CT showed that the lateral lymph nodes were enlarged (arrow) before interventional angiography

On June 21, 2021, ultrasonography indicated the possibility of DVT in the right common iliac vein (Fig. 2), and the right lower limb of the patient was obviously swollen, so it was recommended to remove the thrombus by interventional therapy. Computed tomography (CT) showed suspected soft tissue nodules in the bilateral pelvic wall and no liver or lung metastasis (Fig. 3). The MRI results showed that the pelvic lateral lymph nodes were enlarged (Fig. 1C). The patient’s serum cancer antigen 199 (CA199) level was 37.4 U/ml, and her D-dimer level was 0.63 µg/ml. Other parameters were normal.

**Fig. 4:**
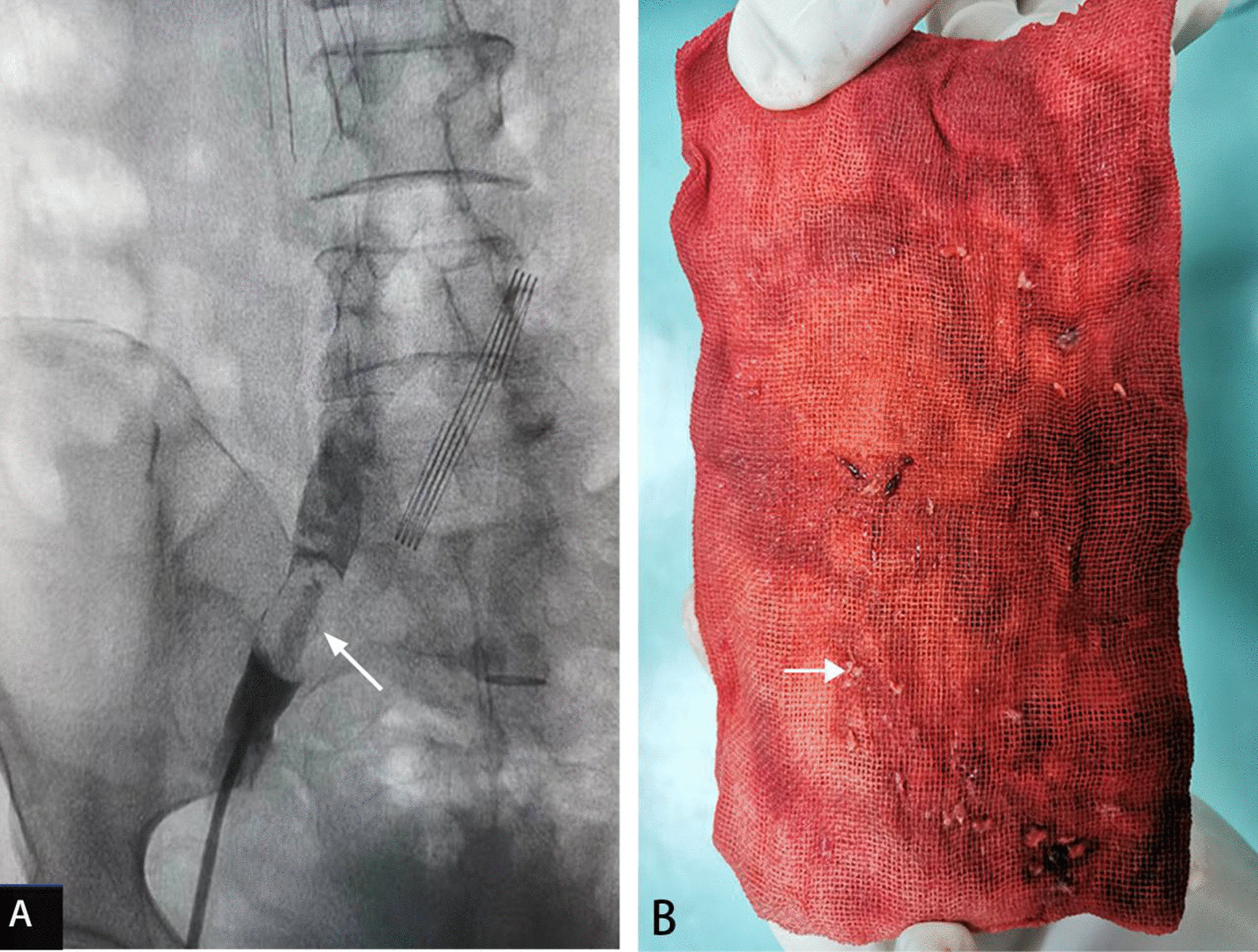
**A** Interventional angiography showed a circular mass in the right common iliac vein (arrow). **B** Biopsy specimen of tumor thrombus (arrow)

**Fig. 5:**
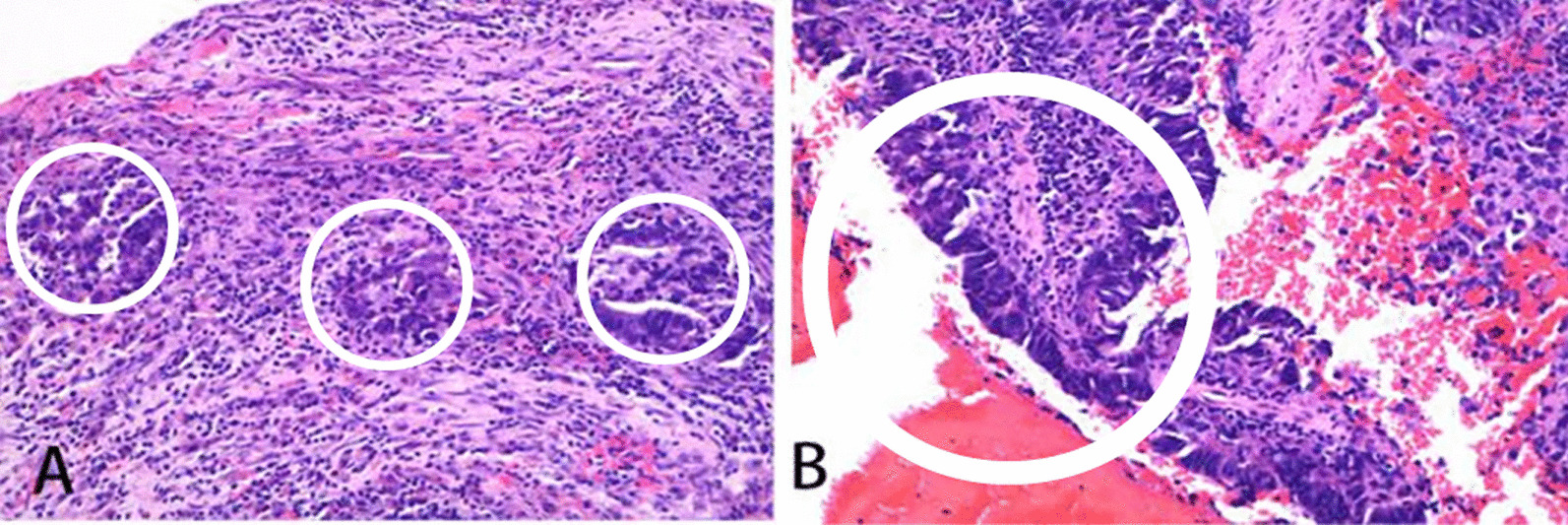
Photomicrographs (hematoxylin and eosin, × 40 magnification). **A** Adenoid structures (white circles) were seen in the fibrous stroma. **B** Nipple-like structures (white circle) were seen in the fibrous stroma

**Fig. 6:**
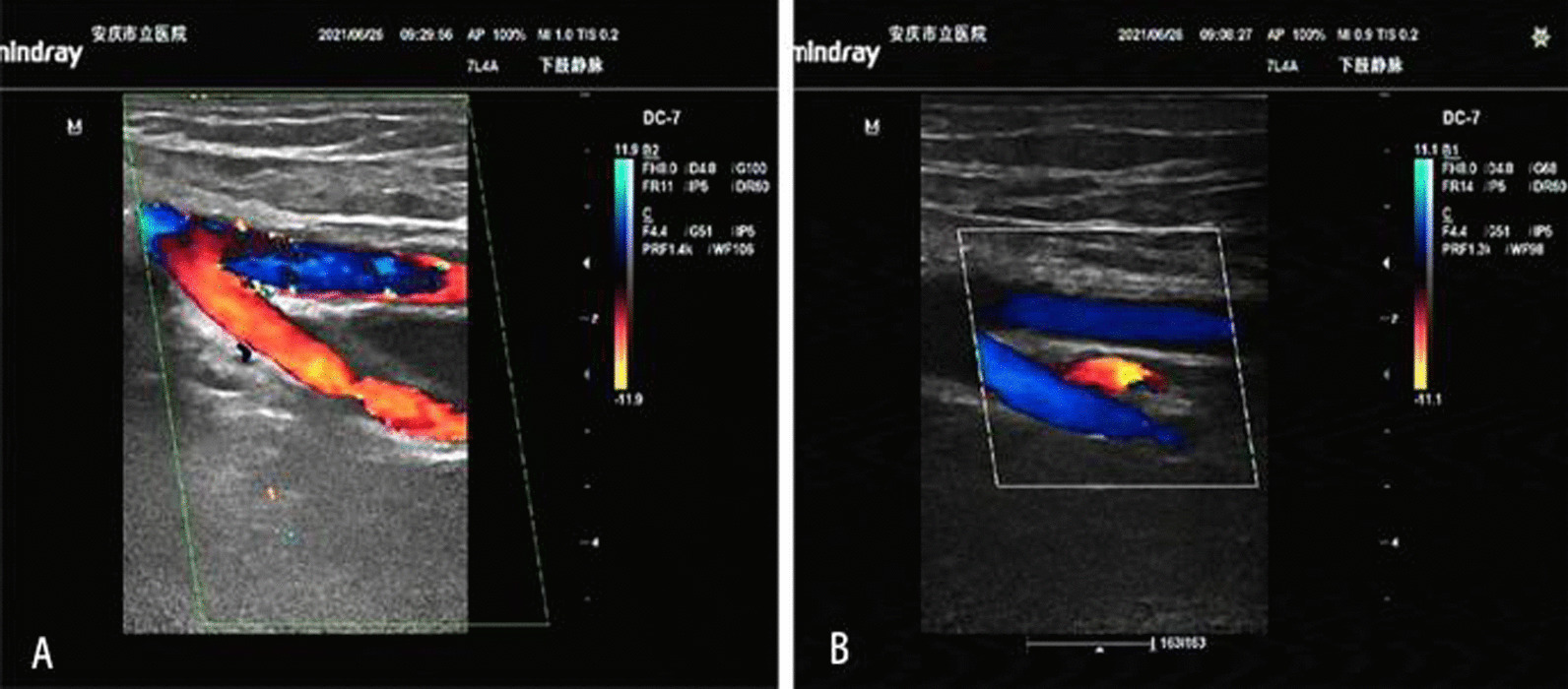
**A**, **B** No tumor thrombus was found by ultrasonography after chemoradiotherapy.

Emergency interventional angiography was performed on June 22, 2021. The results showed that the left femoral vein and iliac vein were unobstructed, as was the deep vein of the right lower limb. However, a circular mass was observed in the right common iliac vein, the boundary was smooth, and the iliac vein cavity was completely blocked (Fig. 4A). IVC angiography plus filter implantation plus balloon dilatation plus aspiration biopsy was performed. A small amount of tissue was biopsied and sent to pathology (Fig. 4B). Photomicrographs showed adenoid and nipple-like structures combined with atypical cells in the fibrous stroma (Fig. 5). Pathology confirmed that the tumor thrombus was metastatic rectal cancer according to the immunostaining results of positive CK20, CDX2 and SATB2. The symptoms of swelling and pain in the right lower leg disappeared after interventional therapy. No tumor thrombus was found in the right common iliac vein by ultrasonography on July 25, 2021 (Fig. 6).

In July 2021, the patient began concurrent chemoradiotherapy, and received capecitabine and radiotherapy. The radiotherapy doses based on the pelvic gross tumor volume (PGTV) and pelvic target volume (PTV) were DT60 Gy/30 f/qd and DT50 Gy/25 f/qd, respectively, and the chemotherapy dose of capecitabine was 2.5 g per day (825 mg/m^2^ twice daily).

**Fig. 7:**
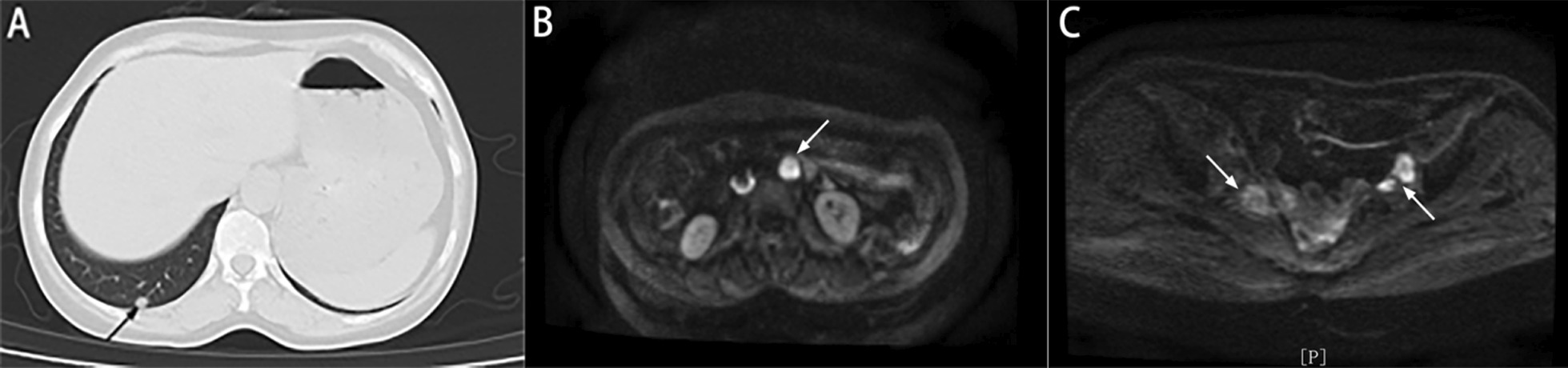
Imaging data of September 2021 **A** CT showed metastatic nodules in the right lung (arrow). **B** MRI showed enlarged lymph nodes in the retroperitoneum (arrow). **C** The pelvic nodules exhibited no obvious change (arrow)

**Fig. 8:**
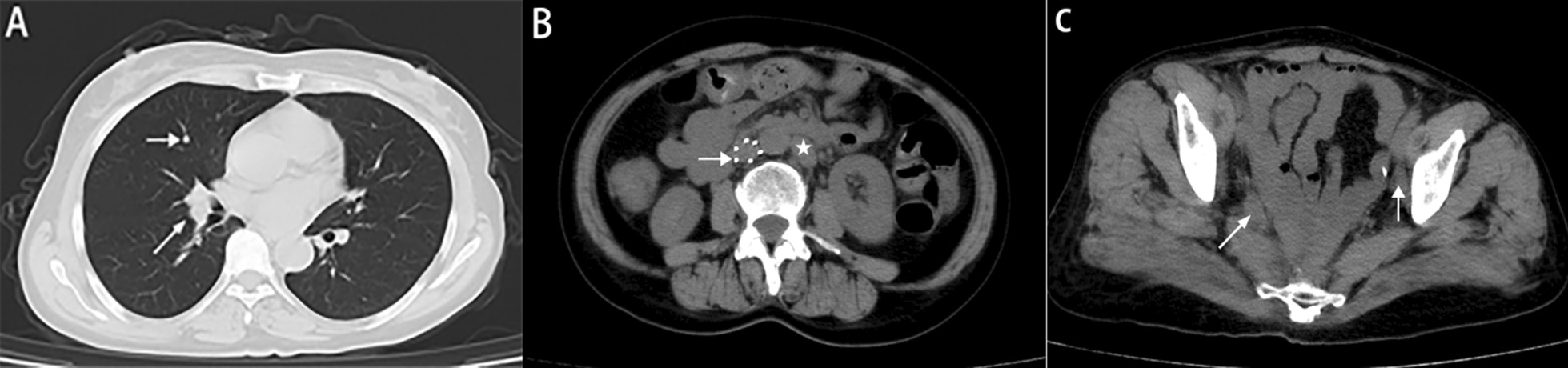
Imaging data from October 2021 **A** CT showed new metastatic nodules in the right lung (arrow). **B** Retroperitoneal enlarged lymph nodes are located on the left side of the aorta (asterisk), and a filter in the Inferior vena cava (arrow). **C** The pelvic nodules were stable (arrow)

**Fig. 9:**
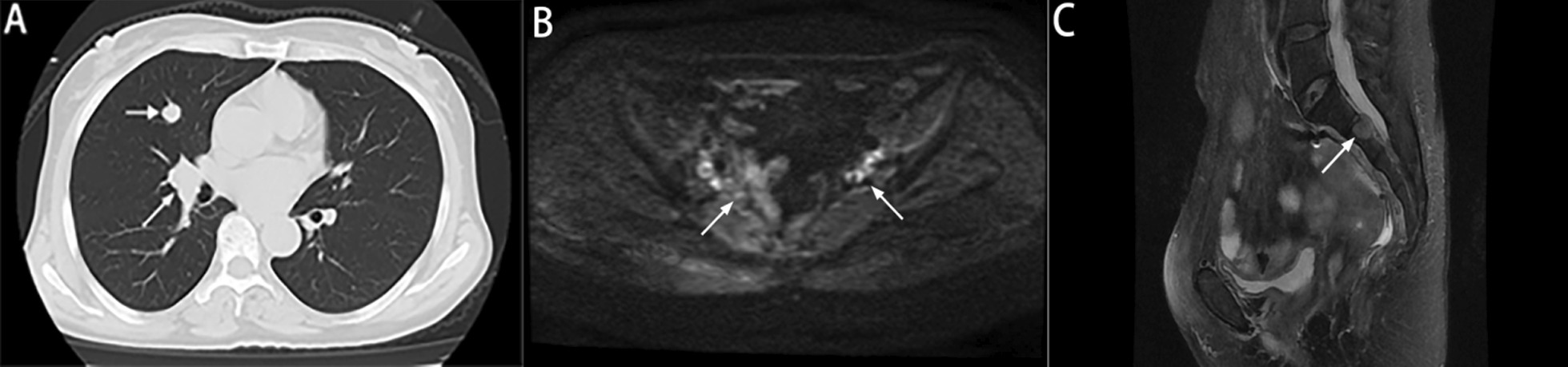
Imaging data from January 2022 **A** CT showed that the right pulmonary nodules had obviously progressed (arrow). **B** The pelvic nodules were significantly enlarged (arrow). **C** MRI showed sacral vertebral metastases (arrow)

On September 9, 2021, the CA199 level was 56.8 U/ml. MRI showed enlarged lymph nodes in the retroperitoneum, and CT showed metastatic nodules in the right lung (Fig. 7). After chemotherapy with irinotecan and capecitabine, the patient developed leukopenia. ECG and ultrasonography showed that cardiac function was decreased, and the patient could not tolerate capecitabine. On October 22, 2021, the CA199 level was 45.4 U/ml, and CT showed that new metastatic nodules of the right lung and the other lesions were stable (Fig. 8). After the bevacizumab + irinotecan + raltitrexed regimen, the patient developed bleeding, diarrhea and fungal infection; thus, the treatment was interrupted again. The patient showed symptoms of abdominal pain on January 5,2022. The CEA and CA199 levels were normal. CT showed that the right pulmonary metastatic nodules had obviously progressed, MRI showed 2^nd^ sacral vertebral metastases, retroperitoneal lesions were stable, and pelvic lymph nodes were significantly enlarged (Fig. 9). Due to the poor physical condition, the patient gave up further treatment, and accepted analgesic therapy. At present, the patients is living with tumors and systemic pain.

## Discussion

An intravascular tumor thrombus is defined as the extension of a tumor into a vessel. Its presence changes the stage, prognosis, and treatment of disease. It occurs in a wide variety of malignancies, most frequently in renal cell carcinoma (RCC), Wilms tumor, adrenal cortical carcinoma (ACC), and hepatocellular carcinoma (HCC) [8–11]. The cause of tumor thrombus formation remains unclear, but the possible theoretical basis is as follows: (1) activation of oncogenes and inactivation of tumor suppressor genes [12]; (2) abnormal protease system in the extracellular matrix [13]; (3) changes in adhesion molecules [14]; and (4) synergistic effect of tumor angiogenic factors [15].

Some scholars have suggested that tumor thrombi from rectal cancer are attributed to pelvic metastatic nodules and can involve either the inferior mesenteric vein (portal venous system) or the internal iliac veins (systemic venous system) [16, 17]. According to a review of the interventional and imaging data, the tumor thrombus identified in the current case was located in the lumen of the common iliac vein, and no metastatic nodules were found outside the cavity, so, we suspected that the tumor thrombus might have originated from the internal iliac vein (pelvic nodules invaded the vessel), and then returned to the common iliac vein (clogged here). Unfortunately, we failed to determine whether there were tumor thrombi in the internal iliac vein. Of note, we hypothesized that the occurrence of the tumor thrombus in this patient was related to the following factors: (1) not only was the pathological stage late, but intravascular tumor thrombus and mesangial tumor thrombus also developed; (2) because the side effects of chemotherapy were too severe, the patient could not bear a sufficient drug dose; (3) neoadjuvant chemoradiotherapy was not performed; and (4) the patient refused radiotherapy after surgery.

After radical proctectomy, right lower limb pain with a skin color change suddenly appeared during follow-up, and the preliminary clinical diagnosis was lower limb DVT. The reason for misdiagnosis was based on the following: (1) in patients with postoperative pelvic lymph node metastasis, tumor secretion of cancerous substances can promote a hypercoagulable state in the blood, leading to DVT, and (2) DVT easily appears in secretory adenocarcinoma, including pancreatic cancer, bronchogenic carcinoma, gastric cancer, colorectal cancer, ovarian cancer and adrenal tumors [18].

The clinical symptoms of tumor thrombi and venous thrombi are very similar, and can be distinguished by imaging and angiography. Both enhanced CT and MRI have high accuracy in showing enhanced tumor trombi, whereas there is no enhancement of venous thrombi. Under contrast-enhanced ultrasound, the activity of a tumor thrombus is enhanced. Vessel expansion and uptake of fluorodeoxyglucose on PET-CT are observed. On angiography, the “streak and thread” sign may be seen. Venography will show filling defects within the affected vessel. Crucially, the diagnosis can be confirmed by aspiration biopsy.

The patient reported here had not received radiotherapy in the past, and the metastatic pelvic nodules could not be resected radically, so concurrent pelvic chemoradiotherapy was adopted. However, after therapy, the patient had lung and retroperitoneal metastasis, so systemic chemotherapy needed to be continued.The primary regimen of systemic therapy was FOFIRI plus bevacizumab (KRAS mutated), but the patient was in an anticoagulant state and could not undergo invasive catheterization, so the regimen of capecitabine plus irinotecan was ultimately preferred.However, after treatment, the patient could not tolerate capecitabine due to severe heart side effects, and the disease progressed. After communicating with the patients, the regimen of irinotecan plus retetrexed plus bevacizumab was used.Unfortunately, the patient had adverse reactions such as bleeding, infection and diarrhea, and the follow-up systemic treatment was interrupted.It has been 12 months since the patient’s diagnosis of tumor thrombus.A study reported that the 2-year survival rate was only 26% in colorectal cancer patients with tumor thrombosis [5].

This case enlightened the authors due to the following observations: (1) For patients with sudden swelling of the lower extremities after proctectomy, we should suspect the possibility of tumor thrombus. Although imaging examinations such as enhanced CT, contrast-enhanced ultrasonography, MRI and PET-CT can distinguish venous thrombi from tumor thrombi, pathological results are the gold standard. During interventional angiography, the patient underwent pathological biopsy and balloon dilatation and prophylactic filter placement, taking comprehensive consideration of treatment and diagnosis. (2) Contrast-enhanced CT, MRI, ultrasonography or PET-CT should be performed before interventional angiography when the patient is suspected of having a tumor thrombus because contrast-enhancement can be used to distinguish venous thrombi from tumor thrombi [19–22].

The incidence of tumor thrombus after rectal cancer surgery is very low, and the incidence of tumor thrombus located in the common iliac vein is even lower. For patients with sudden swelling of the lower extremities after proctectomy, we should suspect the possibility of tumor thrombus.

## Conclusion

Venous thromboembolism is a well-recognized and relatively frequent complication of malignancy, whereas a tumor thrombus is a rare metastasis and indicates a poor prognosis in rectal cancer. The correct diagnosis of a tumor thrombus and its differentiation from venous thromboembolism can aid in decision-making regarding the right treatment plan and prolong the survival of patients.

## Data Availability

Not applicable.

## Abbreviations

APR: Abdominal-perineal resection
DVT: Deep venous thrombosis
CRC: Colorectal cancer
CRM: Circumferential resection margin
MRI: Magnetic resonance imaging
CEA: Carcinoembryonic antigen
CA199: Cancer antigen 199
CT: Computed tomography
PGTV: Pelvic gross tumor volume
PTV: Pelvic target volume
RCC: Renal cell carcinoma
ACC: Adrenal cortical carcinoma
HCC: Hepatocellular carcinoma
PET-CT: Positron emission tomography-computed tomography
IVC: Inferior vena cava
IMV: Inferior mesenteric vein
